# Cysteamine, an Endogenous Aminothiol, and Cystamine, the Disulfide Product of Oxidation, Increase Pseudomonas aeruginosa Sensitivity to Reactive Oxygen and Nitrogen Species and Potentiate Therapeutic Antibiotics against Bacterial Infection

**DOI:** 10.1128/IAI.00947-17

**Published:** 2018-05-22

**Authors:** Douglas J. Fraser-Pitt, Derry K. Mercer, Daniel Smith, Aleksandra Kowalczuk, Jennifer Robertson, Emma Lovie, Peter Perenyi, Michelle Cole, Michel Doumith, Robert L. R. Hill, Katie L. Hopkins, Neil Woodford, Deborah A. O'Neil

**Affiliations:** a NovaBiotics Ltd., Craibstone, Aberdeen, United Kingdom; b Antimicrobial Resistance and Healthcare Associated Infections (AMRHAI) Reference Unit, National Infection Service, Public Health England, London, United Kingdom; University of California, Davis

**Keywords:** Pseudomonas aeruginosa, antibiotic resistance, antimicrobial agents, azithromycin, colistin, cysteamine, innate immunity, nitric oxide, reactive oxygen species, vanin-1

## Abstract

Cysteamine is an endogenous aminothiol produced in mammalian cells as a consequence of coenzyme A metabolism through the activity of the vanin family of pantetheinase ectoenzymes. It is known to have a biological role in oxidative stress, inflammation, and cell migration. There have been several reports demonstrating anti-infective properties targeting viruses, bacteria, and even the malarial parasite. We and others have previously described broad-spectrum antimicrobial and antibiofilm activities of cysteamine. Here, we go further to demonstrate redox-dependent mechanisms of action for the compound and how its antimicrobial effects are, at least in part, due to undermining bacterial defenses against oxidative and nitrosative challenges. We demonstrate the therapeutic potentiation of antibiotic therapy against Pseudomonas aeruginosa in mouse models of infection. We also demonstrate potentiation of many different classes of antibiotics against a selection of priority antibiotic-resistant pathogens, including colistin (often considered an antibiotic of last resort), and we discuss how this endogenous antimicrobial component of innate immunity has a role in infectious disease that is beginning to be explored and is not yet fully understood.

## INTRODUCTION

Cysteamine (CYS) is produced in the body through the cleavage of pantetheine to form CYS and pantothenate (vitamin B_5_) as a breakdown product of coenzyme A (1). CYS itself is highly reactive, and it readily oxidizes in solution to form the disulfide cystamine (CTM) in the presence of oxygen or through Fenton chemistry in the presence of transition metals, releasing free radicals and hydrogen peroxide (2). In a reducing environment in the absence of transition metals, CYS can act as an antioxidant, so its activity is highly dependent upon the physiological context. It will also readily form mixed disulfides with susceptible cysteine sulfhydryl groups in a process called cysteaminylation (3), which is key for many reported biological activities. It may also react with aldehyde groups, which has been suggested as the mechanism behind Ehlers-Danlos syndrome-associated side effects of long-term systemic clinical use (4). Mammalian cells can express aminothiol dioxygenase (ADO), which can oxidize CYS to hypotaurine and taurine (5). Bacterial, fungal, and many eukaryotic parasites (including Plasmodium spp.) cells do not carry the ADO gene, and this may explain selective toxicity. CYS is also a substrate for mammalian vascular adhesion protein 1 (VAP-1), also known as adipocyte copper amine oxidase 3 (AOC3), which can form hydrogen peroxide and ammonia by-products (6, 7). The breakdown of coenzyme A via CYS to taurine is thought to be an underestimated yet significant proportion of sulfur metabolism in mammals (8), but the short-lived nature of CYS and the difficulty of distinguishing it from other small biological thiols have made studying the molecule challenging, and its biological role has likely been underappreciated.

CYS has been licensed for use for >25 years for the treatment of nephropathic cystinosis, depleting cystine from human cells (9) to counteract an inability to remove it from the lysosome (10–12). It has also been investigated for a wide range of other indications, including as a radioprotectant (13) and as a treatment for acetaminophen poisoning (14), Huntington's disease (15), Parkinson's disease (16), and nonalcoholic fatty liver disease (17). CYS is also known to be a broad-spectrum anti-infective (18–20). The oxidized disulfide CTM has been shown to possess antiretroviral properties against HIV (21), and endogenously produced CYS was recently shown to limit influenza virus replication in A549 cells (22). It is also being studied as an adjunct to antimalarial therapy (23, 24). We and others have previously demonstrated that CYS has antimicrobial properties against bacteria associated with cystic fibrosis (CF) respiratory tract infections, including Pseudomonas aeruginosa, Mycobacterium abscessus, and the Burkholderia cepacia complex (BCC). Here, we demonstrate that intravenous (i.v.) or dry-powder inhalation (d.p.i.) administration of CYS potentiates the activities of both ciprofloxacin and tobramycin in neutropenic thigh and lung mouse models of infection, respectively, against P. aeruginosa.

We report here that exposure to CYS in a pro-oxidative environment, or to the oxidized disulfide CTM itself, dysregulates bacterial metabolism and reduces the capacity of P. aeruginosa to resist oxidative and nitrosative stress elicited by exogenous sources. CYS can also readily react with nitric oxide (NO) donors to form a new adduct with characteristics suggestive of *S*-nitrosocysteamine. S-nitrosothiols are a family of compounds with an emerging role in immunity (25), including maintenance of cell barrier integrity against infection and repair (26, 27) and cell signaling (28). CYS also impairs bacterial virulence at subinhibitory levels, with striking effects on bacterial pigment production, including phenazines in P. aeruginosa and pyomelanin in BCC.

Colistin is one of the few remaining options for treatment of infections caused by multidrug-resistant (MDR) or extensively drug-resistant (XDR) strains of Gram-negative pathogens. In the last few years, there have been reports of resistance to colistin in strains of Enterobacteriaceae mediated by plasmid-borne *mcr* genes encoding phosphoethanolamine transferases, initially in China (29) but subsequently from around the world (30–32). Widespread emergence of transferable colistin resistance in clinical cases would pose a serious public health risk. We demonstrate that CYS can potentiate the activity of colistin, including the reversal of clinically defined resistance in clinical isolates and engineered Escherichia coli isolates expressing *mcr-1* and against colistin-resistant Klebsiella pneumoniae (non-*mcr*-mediated) strains in *in vitro* tests. We have also previously demonstrated CYS potentiation of the macrolide azithromycin (33). Azithromycin is also a component of the recommended “last-line” dual therapy for gonorrhea, to which treatment option-limiting resistance has recently emerged (34, 35). Antibiotic resistance in Gram-positive pathogens also remains a major clinical challenge, and we set out to discover if CYS could reverse antibiotic resistance in several methicillin-resistant Staphylococcus aureus (MRSA) strains. We demonstrate the potential for CYS to be repurposed as a short-term-use antibiotic potentiator with broad-spectrum activity in enhancing multiple different classes of antibiotics against a range of antimicrobial-resistant (AMR) pathogens that are of urgent concern to health authorities worldwide.

## RESULTS

### Cysteamine and cystamine sensitize P. aeruginosa to killing by reactive oxygen and nitrogen species.

**FIG 1:**
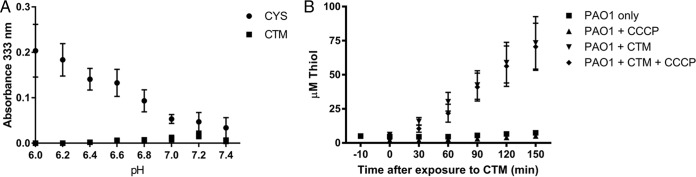
CYS and NaNO_2_ react to form a new product with absorbance peaks at 333 and 545 nm, typical of the S-nitrosothiol *S*-nitrosocysteamine. (A) The reaction favors acidic conditions, as shown by differences in absorbance at 333 nm above background, where 1 mg/ml CYS was reacted with 1 mg/ml NaNO_2_ at 37°C in the dark for 1 h in phosphate-buffered saline across the pH range 6 to 7.4. The disulfide CTM did not react with NaNO_2_ under the same conditions at the same concentrations. (B) CTM is rapidly converted into thiol by P. aeruginosa PAO1 when 1 × 10^9^ CFU/ml were challenged with 3 mM and grown in glucose minimal medium over time, as detected in the culture media by DTNB. The addition of 10 μM CCCP did not prevent conversion of CTM to thiol or export from the cell.

We have previously demonstrated that CYS can potentiate the activity of the fluoroquinolone ciprofloxacin against resistant BCC strains (19). In Table 1, we demonstrate that CYS and CTM consistently, though modestly, potentiate the activity of ciprofloxacin against sensitive P. aeruginosa type strain PAO1 but that they also (particularly CTM) potentiate the antimicrobial activity of the reactive oxygen species (ROS)-generating chemicals paraquat and hydrogen peroxide, to which P. aeruginosa is usually resilient. CYS and CTM also potentiated the antimicrobial activity of the NO donor *S*-nitroso-*N*-acetyl-dl-penicillamine (SNAP). CTM had more pronounced potentiation of the MIC than CYS (Table 1), but CYS formed an adduct species with SNAP and NaNO_2_ that was bacteriostatic in nature. This was evident from a color change (to pink) in microtiter plates used in checkerboard experiments at high concentrations to determine the relative effects on the MIC, and peaks in UV spectroscopy at 333 nm and 545 nm, characteristic of the S-nitrosothiol compound *S*-nitrosocysteamine, although the identity was not confirmed (36). Like other biological S-nitrosothiol compounds, the reaction between the NO donor and thiol was optimal under slightly acidic pH conditions (36) but within physiological parameters (37) for inflamed tissues (Fig. 1). The reaction was thiol specific, as no change in absorbance above background was seen across the same pH range when using the disulfide CTM.

**TABLE 1:** CYS and CTM potentiate the antimicrobial activities of ciprofloxacin and reactive oxygen and nitrogen species; MICs of ciprofloxacin, paraquat, H_2_O_2_, and SNAP versus P. aeruginosa PAO1

Drug	MIC (μg/ml)	
	Alone	In combination
CYS vs. ciprofloxacin		
CYS	256	128
Ciprofloxacin	0.0625	0.0325
CTM vs. ciprofloxacin		
CTM	1,024	256
Ciprofloxacin	0.0625	0.03125
CYS vs. paraquat		
CYS	256	<16
Paraquat	>154.3	77.1
CTM vs. paraquat		
CTM	1,024	64
Paraquat	>154.3	0.3
CYS vs. H_2_O_2_		
CYS	256	256
H_2_O_2_	2.443	1.22
CTM vs. H_2_O_2_		
CTM	1,024	<32
H_2_O_2_	2.443	0.305
CYS vs. SNAP		
CYS	256	128
SNAP	>5	5
CTM vs. SNAP		
CTM	1,024	64
SNAP	>5	0.3125

### Cystamine disrupts P. aeruginosa metabolism, reducing power, and defense against ROS.

We determined that P. aeruginosa PAO1 rapidly exports free thiol in response to treatment with the disulfide CTM by a process that was not inhibited by the ionophore carbonyl cyanide *m*-chlorophenyl hydrazine (CCCP) (Fig. 1) and found that more thiol accumulates in bacterial cells treated with CTM than in cells treated with CYS. The dysregulation of cellular thiols caused by entry of CTM also disrupts P. aeruginosa PAO1 metabolism, as demonstrated by the reduced MIC of the strain when grown in M9 minimal medium with selected tricarboxylic acid (TCA) cycle carbon sources in the presence of CTM in comparison to media containing glucose (Table 2). The MIC in minimal medium with glucose as a carbon source was higher than under standard conditions using Mueller-Hinton broth (1,024 μg/ml), whereas when grown on oxaloacetate, the MIC dropped to 2 μg/ml. The availability of different carbon substrates has a big effect on cystamine toxicity.

**TABLE 2:** Antimicrobial activity of CTM against P. aeruginosa PAO1 is greatly affected by the carbon source^*a*^

Minimal medium carbon source (5 mM)	Median CTM MIC against P. aeruginosa PAO1 (μg/ml) at 20 h
Glucose	1,024
Oxaloacetate	2
Succinate	256
Citrate	128

a The MIC of CTM against P. aeruginosa PAO1 is shown when grown in minimal medium using different carbon sources, including glucose and selected TCA cycle intermediates.

Glucose can be used to replace pools of NADPH, which can be utilized for generating energy but is also required for restoring redox balance in the cell. We discovered that the NADP/NADPH ratio in P. aeruginosa PAO1 is also altered by CTM. When P. aeruginosa PAO1 was grown in RPMI medium with glucose and a subinhibitory concentration (256 μg/ml) of CTM for 20 h at 37°C, the NADP/NADPH ratio was 1.49:1 compared with 1.19:1 for cells treated with vehicle (water) alone. When it was grown without glucose, the NADP/NADPH ratio was 0.66:1 when treated with CTM at 256 μg/ml compared with 0.56:1 with vehicle only (*n* = 3), demonstrating that CTM treatment reduces the cellular NADPH pool.

**FIG 2:**
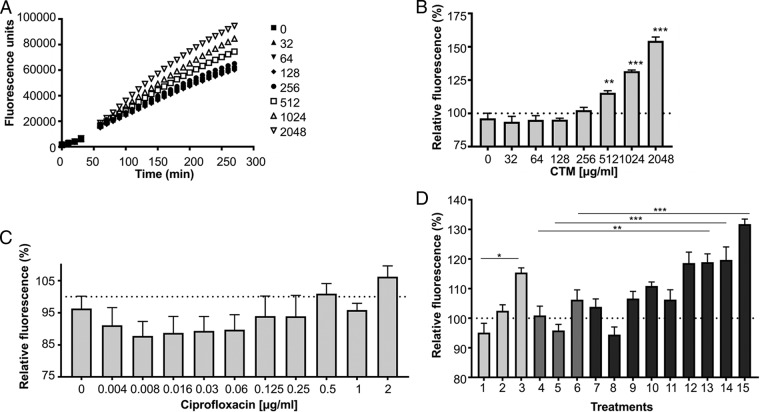
ROS production in P. aeruginosa PAO1 as detected by H2DCFDA fluorescence over time in response to CTM (A) and after 2-h challenge with CTM only (B), ciprofloxacin only (C), or selected combinations of both (D). (D) Bars 1 to 3 show CTM only at 64, 256, and 512 μg/ml. Bars 4 to 6 show ciprofloxacin only at 0.5, 1, and 2 μg/ml. Bars 7 to 9 show CTM at 64 μg/ml with ciprofloxacin at 0.5, 1, and 2 μg/ml. Bars 10 to 12 show CTM at 256 μg/ml with ciprofloxacin at 0.5, 1, and 2 μg/ml. Bars 13 to 15 show CTM at 512 μg/ml with ciprofloxacin at 0.5, 1, and 2 μg/ml (*n* = 3). One-way analysis of variance (ANOVA) with Tukey's *post hoc* analysis (*, *P* < 0.05; **, *P* < 0.01; ***, *P* < 0.001).

Unlike CYS (data not shown), the addition of CTM to Mueller-Hinton broth growth media did not raise the level of ROS above those of controls in uninoculated media, as detected by 2′,7′-dichlorohydrofluorescin diacetate (H2DCFDA) fluorescence. However, when the media were inoculated with P. aeruginosa PAO1 (at a standardized 5 × 10^5^ CFU/ml, the same amount used in MIC determination), the addition of CTM raised the amount of ROS produced relative to uninoculated and untreated controls (Fig. 2). Ciprofloxacin itself is known to induce ROS production, secondary to its activity against DNA gyrase and topoisomerase IV, and this is detectable over time when used in this sensitive strain at concentrations far above the MIC (38), but it was not significant at the concentrations shown using this inoculum size. Interestingly CTM dose-dependently significantly induced ROS formation by P. aeruginosa PAO1 at 512 μg/ml, one doubling dilution below the MIC, from 70 min after exposure onward. Fluorescence relative to background peaked for most treatments at 2 h (see Fig. S1 in the supplemental material), and at that time point, the combination of CTM and ciprofloxacin induced significantly greater amounts of ROS than either treatment alone at the concentrations shown.

### Cysteamine potentiates antibiotic therapy in mouse models of infection.

**FIG 3:**
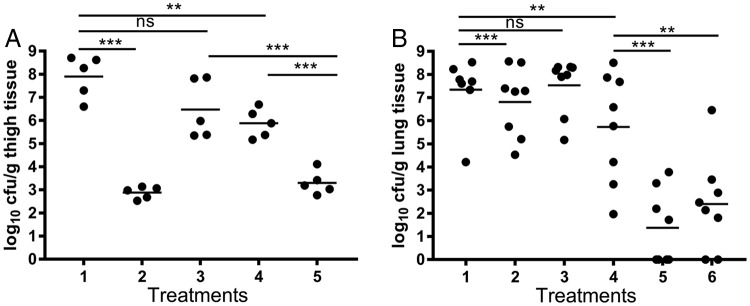
(A) Effects of ciprofloxacin and CYS on the microbial load (expressed as log_10_ CFU per gram of tissue) from the neutropenic mouse thigh model experimentally infected with P. aeruginosa LES431 and treated with vehicle control (lane 1), colistin (5 mg/kg) (lane 2), CYS (1.25 mg/kg) (lane 3), ciprofloxacin (15 mg/kg) (lane 4), and ciprofloxacin plus CYS (lane 5) (*n* = 5 animals per treatment group). (B) Effects of d.p.i.-administered tobramycin and CYS (expressed as log_10_ CFU per gram of tissue) in the neutropenic acute lung model of infection with P. aeruginosa ATCC 27853 treated with vehicle only (3 mg mannitol) (lane 1), 3 mg of 5% CYS (lane 2), 3 mg 10% CYS (lane 3), 0.188 mg tobramycin in lactose (4.5 mg total) (lane 4), 5% CYS plus tobramycin (lane 5), and 10% CYS plus tobramycin (lane 6) (*n* = 8 animals per treatment group). The horizontal lines denote the mean values. One-way ANOVA with Tukey's *post hoc* analysis (ns, not significant; **, *P* < 0.01; ***, *P* < 0.001).

Here, we demonstrate (Fig. 3) that CYS could potentiate the activity of the fluoroquinolone ciprofloxacin in the neutropenic mouse thigh model of infection against the MDR P. aeruginosa Liverpool epidemic strain 431 (LES431), which, unlike PAO1, has a resistant MIC of 4 μg/ml (39) as defined by CLSI and EUCAST clinical breakpoints. Combined i.v. therapy with CYS at 1.25 mg/kg of body weight and ciprofloxacin at 15 mg/kg significantly reduced the microbial burden in the thigh compared with either treatment alone, with a 4.6-log_10_-unit reduction in numbers of CFU per gram. There was no significant difference from a mean 5.02-log_10_-unit reduction in CFU per gram for colistin at 5 mg/kg (used as a positive control). Individually, ciprofloxacin achieved a 2.02-log_10_-unit reduction, and CYS alone elicited a mean reduction of 0.74 log_10_ units, although it was not statistically significant.

Tobramycin is also known to induce ROS formation in BCC (38) and is commonly used clinically as a maintenance therapy in adults and children over 6 years old with cystic fibrosis who are colonized by P. aeruginosa. Therefore, we examined (Fig. 3) the potentiation of tobramycin by inhaled CYS in a neutropenic acute lung P. aeruginosa infection mouse model.

### Cysteamine and cystamine inhibit pigment virulence factors.

The effective dose for potentiating antibiotic activity *in vivo* is below that seen *in vitro* and is achievable therapeutically, so additional factors may be at play. CYS has known immunomodulatory activity (40, 41), which should be considered in an immunocompetent model or host, but it has also been shown to have antivirulence activity (18). While relatively high concentrations of CYS were required for antimicrobial activity *in vitro*, it was noted that subinhibitory concentrations markedly inhibited phenazine production in P. aeruginosa strains (see Fig. S2 in the supplemental material). The phenazine pyocyanin is a redox-active virulence factor with multiple host targets that is implicated in the establishment of lung infection (42, 43). Similarly, pigment secretion in Burkholderia cenocepacia strains was also inhibited. It appears that for B. cenocepacia, CYS may inhibit the polymerization of the secreted homogentisate precursor and inhibit the secretion of pyomelanin from the cell, as the pigment could be released from lysed pellets (see Fig. S3 in the supplemental material). Pyomelanin pigment, detected by absorbance at 480 nm (44), was significantly inhibited by CYS at concentrations above 8 μg/ml. The inhibition of these virulence determinants at sub-MICs may have further benefit therapeutically to enhance cotherapies and assist the innate and adaptive immune responses to infection.

### Potentiation of colistin antimicrobial activity.

We have also explored whether CYS has efficacy for potentiating antibiotics against pathogens beyond the context of cystic fibrosis. We demonstrated that the transformation of a laboratory strain of E. coli (NEB Express) with a plasmid-borne *mcr-1* gene confers CLSI-defined resistance to colistin. Our transformant was resistant to colistin (MIC = 8 μg/ml) compared with the strain containing the empty vector, which was susceptible to colistin (MIC = 2 μg/ml). We showed (Table 3) an 8-fold reduction in the MIC of colistin when tested in combination with CYS against the transformant in checkerboard studies, with the MIC reduced from 8 μg/ml (defined as resistant) to 1 μg/ml (susceptible). The MIC for the strain harboring the empty vector was also consistently reduced slightly, by 2-fold, from 2 μg/ml to 1 μg/ml, which confirms that the effects of CYS are not specific to the *mcr-1*-mediated resistance mechanism.

**TABLE 3:** CYS can potentiate colistin and azithromycin *in vitro*; MICs of colistin and azithromycin with and without CYS against a selection of pathogens^*a*^

Antibiotic	Bacterial species, strain, and genotype or phenotype	Median MIC (μg/ml)^*b*^		% reduction in effective dose
		Without CYS	With CYS	
Colistin	E. coli NEB pET-29B	2 (S)	1 (S)	50
	E. coli NEB pET-29B *mcr-1*	8 (R)	1 (S)	87.5
	E. coli RH14000226 *mcr-1*	4 (R)	2 (S)	50
	K. pneumoniae NB01216 *bla*_NDM-1_, *mgrB*′ (70Ins_IS*5*-like)	64 (R)	16 (R)	75
	K. pneumoniae NB02216 *bla*_KPC-2_, *mgrB*′ (7A→T)	32 (R)	1 (S)	96.9
Azithromycin	S. aureus SACF636 (MRSA)	0.5 (S)	0.25 (S)	50
	S. aureus SACF652 (MRSA)	>256 (R)	0.25 (S)	>99.9
	S. aureus SACF662 (MRSA)	>256 (R)	0.5 (S)	>99.8
	S. aureus SACF667 (MRSA)	>256 (R)	0.5 (S)	>99.8
	S. aureus SACF660 (MSSA)	0.5 (S)	0.25 (S)	50
	S. aureus SACF661 (MSSA)	1 (S)	0.5 (S)	50
	S. aureus SACF663 (MSSA)	128 (R)	0.25 (S)	99.8
	S. aureus SACF665 (MSSA)	0.5 (S)	0.25 (S)	50
	S. aureus SACF666 (MSSA)	1 (S)	0.5 (S)	50
	S. aureus DSM 11729 (MRSA)	16 (R)	1 (S)	93.8
	S. aureus BAA-1717 (MRSA)	8 (R)	4 (I)	50
	N. gonorrhoeae NB04916	32	4	87.5
	N. gonorrhoeae NB03916	>256	>256	0

a Including the interpretive criteria where possible when used alone and in combination.

b S, susceptible; I, intermediate; R, resistant.

We have also demonstrated resistance breaking by CYS in a clinical strain of E. coli, RH14000226, harboring the *mcr-1* gene (confirmed by PCR). E. coli RH14000226 is a sequence type 457 (ST457) isolate harboring the extended-spectrum beta-lactamase CTX-M-27 and the colistin resistance gene *mcr-1*, the latter of which is located on the IncHI2 plasmid 15 (Table 3).

CYS potentiated the activity of colistin against two resistant K. pneumoniae strains that do not carry *mcr-1*. Isolate NB02216 belongs to ST258 and harbors the KPC-2 carbapenemase, while NB01216 belongs to ST14 and harbors the NDM-1 carbapenemase. In both isolates, resistance to colistin was due to inactivation of *mgrB*: in NB02216 via insertion of an IS*5*-like transposase at nucleotide position 70 of *mgrB* and in NB01216 due to an A→T nucleotide substitution at nucleotide position 7 of *mgrB*, resulting in an early stop codon. In the ST258 strain, we demonstrated full restoration of clinical susceptibility to colistin (a 32-fold reduction in the MIC to 1 μg/ml) in the presence of CYS; although the ST14 strain remained colistin resistant, CYS reduced its colistin MIC by 4-fold (Table 3).

### Potentiation of macrolide antimicrobial activity.

We demonstrated considerable synergy between CYS and the macrolide azithromycin against S. aureus. The MICs for five azithromycin-resistant isolates (four MRSA strains and one methicillin-susceptible S. aureus [MSSA] strain) were reduced 16- to 1,024-fold, in each case to below the CLSI and EUCAST clinical susceptibility breakpoints (Table 3); in contrast, the MICs for five azithromycin-susceptible S. aureus strains (one MRSA and four MSSA) were reduced only 2-fold in the presence of CYS.

**FIG 4:**
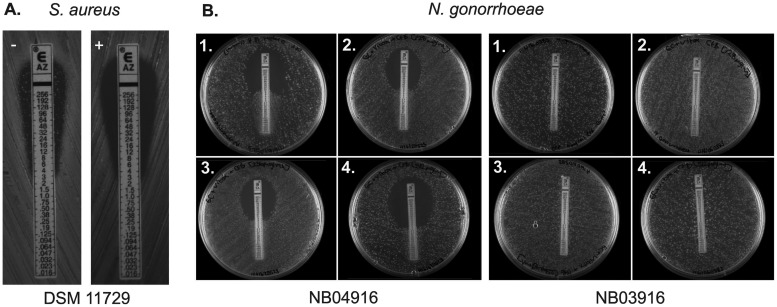
(A) The incorporation of 512 μg/ml CTM in CA MHA removes a subpopulation of resistant cells of S. aureus DSM 11729 cultured with azithromycin Etest strips (control plate [left]). (B) An increased zone of clearance of N. gonorrhoeae strain NB04916 can be observed surrounding azithromycin Etest strips on plates with increasing concentrations of cysteamine (1, 0 μg/ml; 2, 128 μg/ml; 3, 256 μg/ml; 4, 512 μg/ml) incorporated into GC (plus Vitox supplement) agar, whereas there was no apparent difference for strain NB03916.

When azithromycin Etest strips were used to determine the MIC, it was noted that the appearance of a resistant subpopulation of colonies that grew close to the Etest strip in resistant strains was abolished by the incorporation of CTM into the cation-adjusted Mueller-Hinton agar (CA MHA) plates (Fig. 4).

CYS also potentiated the activity of azithromycin against an MDR strain of Neisseria gonorrhoeae, strain NB04916 (Fig. 4), reducing its MIC from 32 μg/ml to 4 μg/ml (Table 3). While this did not restore clinical susceptibility as defined by EUCAST (MIC < 0.5 μg/ml), the 87.5% reduction may still be of clinical value and will be investigated further. This isolate has the −1A deletion in the 13-bp inverted-repeat region between positions −10 and −35 of the *mtrR* promoter, as well as D79N, T86A, and H105Y mutations. In contrast, the addition of CYS did not demonstrably alter the MIC for an N. gonorrhoeae strain in which high-level azithromycin resistance is mediated through the mutation A2059G in all 23S rRNA alleles.

CYS did not potentiate beta-lactam-containing antibiotics against all the bacteria we have tested so far in the *in vitro* systems that we employed (data not shown). Indeed, there was concentration-specific antagonism with all classes of antibiotics containing the beta-lactam ring that we have tested to date. Suspecting that these findings (which contradicted previous CF clinical observations) were specific to beta-lactam/CYS chemistry within the limitations of *in vitro* systems, we investigated if CYS reacts with nitrocefin in bacterial growth media. Hydrolysis of the beta-lactam produced a red product that was rapidly detectable when combined with CYS (data not shown). CTM itself did not react.

## DISCUSSION

**FIG 5:**
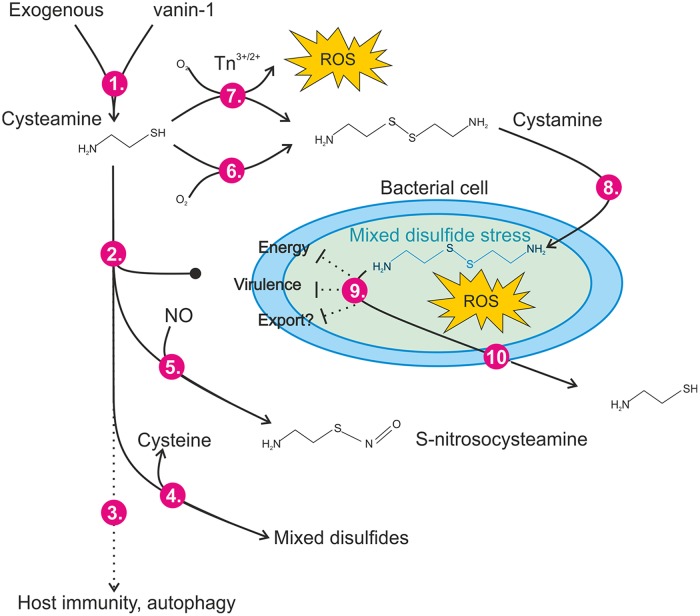
Redox-dependent anti-infective mechanisms of action for cysteamine/cystamine. (Step 1) Cysteamine can be supplied therapeutically or produced endogenously through the action of vanin-1 pantetheinase. (Steps 2 and 3) Cysteamine itself does not pass the bacterial cytoplasmic membranes of rapidly dividing cells (step 2) but is reported to have impacts upon host immunity to infection and autophagy (step 3). (Steps 4 and 5) Cysteamine can react (reversibly) with susceptible cysteine residues in a process termed cysteaminylation (step 4), and we demonstrated that it can form adducts with reactive nitrogen species, possibly forming *S*-nitrosocysteamine in mildly acidic environments (step 5). (Steps 6 and 7) Cysteamine readily forms the disulfide cystamine (and water) in the presence of oxygen (step 6) in a temperature-dependent manner and at millimolar concentrations rapidly generates ROS in the presence of transition metal ions (step 7). (Steps 8 and 9) Cystamine itself may interact with unknown periplasmic targets in Gram-negative cells and can enter the bacterial cell (step 8) via an unknown mechanism, where it generates ROS and interacts with susceptible intracellular targets, leading to dysregulation of small thiol pools and metabolism (step 9), disrupting pigment production or export. (Step 10) Reduced thiols, probably including cysteamine, are exported via an unknown mechanism.

The antimicrobial activity of CYS is mediated by a complex interplay of the redox environment; conversion to CTM; access to the cell membrane or cytoplasm (which is itself likely dependent upon microbial substrate availability); and the chemistry of the site, such as the presence of transition metals, nitrosating species, or other compounds that may react with CYS (Fig. 5). The rate of enzymatic, or chemical, removal of CYS and CTM from the bacteria or host environment is likely to be another factor, which has not been fully explored here. This complexity means that conventional (45) *in vitro* antimicrobial susceptibility test (AST) methods cannot fully capture the utility (or otherwise) that this compound may have as an anti-infective or as an adjunct potentiator of antibiotics. Although we and others have demonstrated direct broad-spectrum antimicrobial activity of CYS (18–20), the concentrations required for microbicidal activity in standardized tests are much higher than those required to achieve antimicrobial/potential activity *in vivo*, and effects upon host cellular immunity cannot be ruled out. Studies (40, 41) have demonstrated that CYS enhanced the microbicidal clearance of P. aeruginosa and BCC from experimentally infected macrophages bearing the F508del cystic fibrosis transmembrane conductance regulator (CFTR) mutation, attributed to reduced proteostasis and enhanced autophagy. Clearly these effects are not relevant to neutropenic wild-type animal studies, but we do not yet have a full understanding of the impact of CYS on the immune response to infection.

Modification of conventional antibiotic susceptibility tests can bridge this MIC gap. For example, we have demonstrated that repeated low dosing (at physiologically achievable levels) *ex vivo* in sputum is antimicrobial (33) against the bacterial load in CF sputum. Substrate availability is important to bacterial sensitivity to CYS, and we are also investigating the effects of the growth phase and aeration upon the MICs of CYS and CTM. Our results suggest CYS might disrupt the TCA cycle in P. aeruginosa, where we achieved particularly low MIC values when grown in oxaloacetate M9 minimal medium. Some of these factors appear to explain the disparity between the relatively high *in vitro* MIC and the lower effective dose in animal studies and human trials. The context of the infection might be critical to the impact of CYS/CTM upon it. Interactions and access to immune mediators also vary from infection to infection.

The potential interplay of CYS and other innate immune effector molecules, such as NO, is fascinating. *S*-Nitrosocysteamine has been considered a potential NO carrier (36), but it was thought to be too short-lived a molecule for use therapeutically. The identity of this adduct needs to be confirmed with nuclear magnetic resonance (NMR) spectroscopy. Whether the species does form endogenously during infection or inflammation needs to be investigated further, though we can detect its formation at physiological pH (though acid pH promotes formation) and are investigating the ability of cysteine, via transnitrosation, to accelerate the production and removal of the species.

There is widespread tissue distribution of vanin-1, including gut, lung, and liver, and differing redox environments, including pH and partial O_2_ pressure (pO_2_), at different body sites. It is therefore possible that expression of the same biological thiol might have different impacts on physiology (including innate immunity) in the lung, for example, compared with the gut. The same might be said for inflamed and healthy tissue. It would also be very interesting to examine the effect of a pro-oxidative insult upon the usually anaerobic environment of the gut (for example, caused by injury) and any role CYS might play in inflammation, immunity, or repair, as vanin-1 is highly expressed there. The *vnn-1* knockout mouse is known to be protected from intestinal inflammation (46, 47), but so far, the only study relating to this mouse knockout and immunity to bacterial infection (to our knowledge) was an investigation into the role of *vnn-1* in granuloma formation and macrophage activation in response to Coxiella burnetii (48). For that pathogen, there was no difference in the ability of the mutant to eliminate infection, but there were impairments in leukocyte recruitment and macrophage-driven antimicrobial activity. Given the potential interaction with the CYS product of vanin-1 and nitric oxide shown in this study, it is interesting that the *vnn-1* knockout had impaired iNOS expression in response to C. burnetii infection.

Studies examining vanin-1 discuss the potential interaction of CYS with the glutathione (GSH) pool of the host (46, 49). Indeed *vnn-1* mice themselves have been demonstrated to be less susceptible to oxidative injury by paraquat than wild-type mice (46), which has been attributed to inhibition of gamma-glutamylcysteine synthetase (γGCS), which is responsible for *de novo* synthesis of GSH, by CTM. Studies examining the toxicity of CYS to human leukocyte lines also demonstrated inhibition of mammalian glutathione peroxidase (2), and glutathione transferases with peroxidase activity are widely expressed in aerobic bacterial pathogens (50), though it is not yet known if this activity is also inhibited by CYS. Although synthesis of GSH has not been studied here, the activity of E. coli γGCS has been shown to be resistant to CTM (51). It is known that the disulfide cystine, as is seen here with CTM, sensitizes bacteria to oxidative stress (52) and that mammalian and bacterial cells respond differently to the disulfide (53). Cystine import systems that are widely conserved in other bacteria, including P. aeruginosa, have been reported in E. coli and have been described as part of the cysteine/cystine futile cycle, a vulnerability in bacterial metabolism that leads to overimport of cystine and GSH-dependent reduction back to cysteine, much of which is exported and then (in a sufficiently oxidative environment) oxidized to cystine again, completing a cycle (52, 54). We demonstrate that thiol export in response to CTM is not dependent upon proton motive force, but whether the import/export mechanisms for CTM/CYS are the same as for cystine/cysteine is not yet known. Early observations in Streptococcus mutans (55) showed that treatment with the disulfide also led to an accumulation of CYS in the culture medium. We suggest that CYS enters the cell as CTM in a Trojan horse-like manner before being reduced back to CYS, or perhaps other thiols, in the cytoplasm, whereupon it is rapidly excreted in a manner similar to that described for cystine. There is then a window of opportunity for CYS to gain access to cytoplasmic cysteine (thiol) targets, including the small thiol pool (such as GSH or bacilithiol in Gram-positive species) or iron-sulfur clusters, which might be sensitive to oxidation or cause damage via Fenton chemistry with intracellular transition metal ion stores (2), and this window may be wider depending upon the energy status and substrate availability to the cell. This certainly agrees with studies conducted in our laboratory comparing glucose-containing and glucose-free media, which demonstrated lower MICs in the absence of glucose.

The ability of CYS to sensitize bacteria to oxidative stress by depleting cellular reductants is one mechanism that can explain the broad-spectrum potentiation of different classes of antibiotics that are known to induce ROS formation in bacterial cells, and indeed, the addition of exogenous catalase raises the MIC of CYS marginally (data not shown); however, we believe it is not the only mechanism at work. We can potentiate azithromycin in both resistant Gram-positive S. aureus and Gram-negative N. gonorrhoeae, whose resistance is mediated by very different mechanisms, and azithromycin is a known antioxidant (56) that did not induce ROS formation in our assays. The azithromycin-potentiating activity of CYS against N. gonorrhoeae strain NB04916, where mutations in the *mtrR* gene and promoters are likely to mediate efflux-driven resistance (57), contrasts with the lack of effect in NB03196, which suggests an ability to inhibit efflux of the antibiotic. Similarly, the antimicrobial action of colistin does not involve ROS formation (58). Multiple effects on metabolism, virulence, and biofilm formation also suggest this is not the only mechanism, and a number of potential targets are being investigated.

The antibiofilm formation effects of CYS have been reported previously (18), but here, we demonstrate for the first time the inhibition of phenazine production in various type and clinical strains of P. aeruginosa. Phenazines, such as pyocyanin, are redox-active antimicrobial virulence factors that have been demonstrated to have a detrimental effect on host immune defenses (42, 43). A previous study examining the function of the Pseudomonas quinolone signal (PQS) biosynthesis pathway identified cysteamine phosphate as a substrate for PqsE, a protein that plays an incompletely defined role in the PQS quorum-sensing regulation of a range of virulence factors, including phenazine production (59). It is not known at this stage if CYS modification of this target, or via competitive inhibition, is responsible for the reduction in pigment production seen after sub-MIC exposure, but it is being investigated. It is interesting that CYS also inhibited pyomelanin synthesis and/or secretion in B. cenocepacia (see Fig. S2 in the supplemental material). Pigment production in B. cenocepacia is also implicated in resistance to host-mediated oxidative defense and intracellular survival (60). The synthesis of the pigment is distinct from phenazine synthesis in P. aeruginosa, though some P. aeruginosa strains do produce it, as well as a range of other pathogens, and it is thought to contribute to persistence in CF airways. The pigment is exported from the cell via the ABC transporter HatABCDE (61), and retention of pyomelanin within the B. cenocepacia pellet again points to inhibition of ATP-driven export.

Although there is still a great deal of work to be done to fully unravel and calibrate the role of CYS in immunity to infection *in vivo*, it is important for us now to consider CYS as an endogenous active sulfur species alongside the much better known reactive oxygen and nitrogen species. Our present study highlights the potential for CYS to enhance the antimicrobial activity of therapeutic antibiotics against pathogens, for which the emergence of resistance is a growing concern that limits current clinical options (62, 63). Understanding how the physiological context impacts CYS activity will help to inform the choice of the target infection in any future developments for repurposing CYS as an adjunct to antimicrobial therapy, but our data thus far clearly demonstrate that it has possible benefits for the treatment of pulmonary infections in cystic fibrosis patients and far beyond.

## MATERIALS AND METHODS

Unless otherwise stated, chemicals, reagents, and media were purchased from Sigma-Aldrich (St. Louis, MO, USA).

### Bacterial strains and culture conditions.

The commercially available E. coli NEB Express strain was chosen for transformation with *mcr-1*, as the laboratory strain was shown to be susceptible to colistin and is designed to maximize transformation efficiency and to express recombinant proteins. The clinical MDR E. coli RH14000226 strain was confirmed as colistin resistant. Two MDR K. pneumoniae strains (NB01216 and NB02216) were also chosen for checkerboard analysis alongside CYS due to their colistin susceptibility profiles and clinical significance. For the colistin antimicrobial susceptibility experiments, all the strains were screened for carriage of the *mcr-1* gene, using the primers mcr-1_for (TCCAAAATGCCCTACAGACC) and mcr-1_rev (GCCACCACAGGCAGTAAAAT).

Eleven strains of S. aureus were examined, including 6 defined as MRSA by selective isolation on mannitol salt agar with oxacillin and profiling of susceptibility to oxacillin. Nine strains were clinical isolates, and two were MRSA type strains. Seven out of the 11 strains were shown to be resistant to azithromycin using Etest screening and CLSI standardized MIC broth microdilution experiments (see below).

Two MDR strains of N. gonorrhoeae (NB03916 and NB04916) were chosen for experimentation based upon clinical importance and susceptibility profiling.

The well-characterized P. aeruginosa type strain PAO1 was used for *in vitro* antimicrobial mechanism-of-action studies. The MDR LES431 strain of P. aeruginosa was chosen for the mouse thigh model of infection, and P. aeruginosa ATCC 27853 was the established strain used in the mouse acute lung model of infection. A selection of CF isolates were also tested for phenazine production, as were pigmented type and clinical strains of B. cenocepacia.

All bacteria were grown in Mueller-Hinton broth (with cation modification), except N. gonorrhoeae strains, which were grown on GC agar plus Vitox supplement (Oxoid, Thermo Scientific, United Kingdom). The strains were maintained with the addition of the appropriate antimicrobial agents where required, although they were withdrawn prior to overnight subculture for antimicrobial susceptibility testing with no detectable loss of plasmids or cloned inserts.

MIC broth microdilution experiments (see below) were also conducted in M9 minimal medium supplemented with different carbon sources at 0.4% (wt/vol) and corrected to pH 7.0 to examine the impacts of the different carbon sources on the antimicrobial activity of cystamine. M9 minimal medium consisted of 50 mM Na_2_HPO_4_ · 7H_2_O, 22 mM KH_2_PO_4_, 18.7 mM NH_4_Cl, 8.5 mM NaCl, 2 mM MgSO_4_, and 0.1 mM CaCl_2_. Carbon sources, including glucose, oxaloacetic acid (oxaloacetate), sodium succinate dibasic hexahydrate (succinate), and sodium citrate dihydrate (citrate), were added to 0.4% (wt/vol) from filter-sterilized stock solutions.

### Determination of thiol content using DTNB.

P. aeruginosa PAO1 was grown overnight at 37°C on Mueller-Hinton agar prior to inoculation of 19 ml prewarmed glucose minimal medium with 1 ml of 2 × 10^12^ CFU suspended in phosphate-buffered saline (PBS) in a sterile flask and incubated with shaking at 150 rpm for 4 h to reach logarithmic growth phase. Ten minutes prior to exposure to CTM, selected cultures were treated with 10 μM CCCP to collapse the proton gradient. The background thiol contents of the supernatants were also determined by removing 1 ml culture and pelleting the bacteria via centrifugation at 2,350 × *g* for 5 min. Dithionitrobenzoic acid (DTNB) was added to the clarified supernatant to a final working concentration of 0.1 mM per 0.1-ml volume (from a 20× stock containing 2 mM DTNB and 50 mM sodium acetate) in a 96-well microtiter plate and incubated for 10 min at room temperature prior to reading at 410 nm on Biotek plate readers. Reduced solutions of cysteine were used as standards for concentration determination. Background controls for CCCP, CTM, and medium alone were also determined. Cellular thiol contents were determined by mechanical disruption using ZR Bashing Bead lysis tubes for 5 min prior to DTNB assay.

### Detection of ROS using H2DCFDA.

H2DCFDA is a cell-permeable nonfluorescent compound that reacts with reactive oxygen species to form the highly fluorescent 2′,7′-dichlorohydrofluorescein. P. aeruginosa PAO1 at 5 × 10^5^ CFU/ml was challenged with increasing concentrations of ciprofloxacin and CTM dihydrochloride in a checkerboard format, as described previously (64), in 100-μl volumes on a microtiter plate with the addition of H2DCFDA at 1 μM working concentration, and fluorescence was detected over time during incubation at 37°C at excitation/emission wavelengths of 485/20 and 528/20 nm.

### NADP/NADPH ratio determination.

P. aeruginosa PAO1 cells were grown in defined RPMI medium with or without glucose at 2 g/liter for 20 h at 37°C and treated or not with cystamine dihydrochloride as described previously, and the NADP/NADPH ratio was determined colorimetrically at 450 nm using a microtiter assay kit (Sigma-Aldrich) according to the manufacturer's instructions, including the removal of enzymes that may utilize NADPH by passage of clarified lysate through 10-kDa-cutoff filters (Amicon, Merck, Kenilworth, NJ, USA).

### Molecular biology.

The open reading frame sequence for the gene *mcr-1*, a putative phosphoethanolamine transferase (UniProt accession number A0A0R6L508) was synthesized using the GeneArt gene synthesis service (Thermo Fisher Scientific, Waltham, MA, USA). The sequence was amplified by PCR with flanking cloning primers (clon_for, ATTCCATATGATGCAGCATACTTCTGTGTGGTACCG, and clon_rev, TGTACTCGAGGCGGATGAATGCGGTG) to introduce restriction enzyme sites (underlined). They were digested with appropriate restriction enzymes (NdeI and XhoI) and ligated, in frame, into the multiple-cloning site of plasmid pET29b. Plasmids with and without an *mcr-1* insert were transformed into the E. coli NEB Express laboratory strain of E. coli. Internal detection primers (described above) were used to confirm the presence or absence of the *mcr-1* insert in the transformed cells, and expression of the *mcr-1* gene was confirmed due to phenotypic change in the colistin MIC for the strain (using the method described below).

### Antimicrobial susceptibility MIC and checkerboard experiments.

The MIC and minimal bactericidal concentration (MBC) were determined versus CYS and other antibiotics in this study by the method described previously using the CLSI M07-A10 broth microdilution procedure (45). Checkerboard assays of CYS, CTM, and the antibiotics were conducted (64) to assess the combinations of CYS and CTM with ROS- and NO-generating compounds. Definitions of susceptibility or resistance were determined using EUCAST clinical breakpoints for bacteria (http://www.eucast.org/clinical_breakpoints/).

### Etest MIC determination for S. aureus on CA MHA and for N. gonorrhoeae strains on GC agar plates.

Azithromycin-impregnated Etest strips were placed upon CA MHA or GC agar (plus Vitox supplement) containing different concentrations (0 to 512 μg/ml) of CYS, as indicated, inoculated with suspensions of S. aureus or N. gonorrhoeae, respectively, according to the manufacturer's instructions (bioMérieux, France), and incubated for 24 h at 37°C for S. aureus strains and for 48 h at 37°C in a 5% CO_2_ atmosphere for N. gonorrhoeae strains.

### Procedures.

Animal studies were conducted by Evotec (U.K.) Ltd. (mouse acute lung model) and Eurofins Panlabs, Taipei, Taiwan (mouse thigh model). All procedures were performed in accordance with appropriate regulatory licenses held by the entities/personnel who performed the studies.

### Mouse acute lung model of infection.

Male CD1 mice were immunosuppressed/preconditioned with 200 mg/kg and 150 mg/kg cyclophosphamide at 4 and 1 days prior to the study, respectively. CYS and tobramycin were prepared for inhalation formulation in a mannitol vehicle. An infection was established with P. aeruginosa ATCC 27853, with an inoculum of 5 × 10^6^ CFU/ml administered intranasally in a volume of 40 μl following anesthetization with a ketamine/xylazine anesthetic cocktail for 15 min. The treatments were administered approximately 10 min after infection. All treatments and the mannitol vehicle control were administered using a DP-4M insufflator device (Penn-Century, Inc., Philadelphia, PA). The lung tissue burden of each animal, at the clinical endpoint of 24 h postinfection, was determined. The lungs were homogenized in 2 ml PBS, serially diluted in PBS, and plated on Pseudomonas selective agar before quantification after 24 to 48 h at 37°C.

### Mouse thigh model of infection.

Mice were rendered neutropenic with cyclophosphamide prior to infection as described above for the acute lung model of infection. They were separated into groups of 5 animals per treatment. Inoculation (confirmed by culture) was with 1.52 × 10^6^ CFU/ml of P. aeruginosa strain LES431 conducted 1 h prior to treatment, which was i.v. for saline vehicle control-, ciprofloxacin-, and CYS-treated mice and subcutaneous for colistin (used as a positive control). The animals were sacrificed 25 h postinfection (24 h posttreatment), and thigh weights and numbers of CFU per gram of tissue were calculated and recorded.

## Supplementary Material

Supplemental material
