# Supplementary material for: Cysteamine, an Endogenous Aminothiol, and Cystamine, the Disulfide Product of Oxidation, Increase Pseudomonas aeruginosa Sensitivity to Reactive Oxygen and Nitrogen Species and Potentiate Therapeutic Antibiotics against Bacterial Infection
Source: Infect Immun. 2018 May 22;86(6):e00947-17. doi: 10.1128/IAI.00947-17 (PMC5964511; doi:10.1128/IAI.00947-17)
Supplement: Supplemental material [file supp_86_6_e00947-17__index.html]

Supplemental material 

# Cysteamine, an Endogenous Aminothiol, and Cystamine, the Disulfide Product of Oxidation, Increase Pseudomonas aeruginosa Sensitivity to Reactive Oxygen and Nitrogen Species and Potentiate Therapeutic Antibiotics against Bacterial Infection

## Supplemental material

- Supplemental file 1 -

  Fig. S1. Relative H2DCFDA fluorescence of *Pseudomonas aeruginosa* PAO1 cultures treated with different combinations of ciprofloxacin and cystamine as shown over time compared to background fluorescence in media from which data at 2 h is analyzed as shown in Fig. 2. Fig. S2. Addition of sub-MIC concentrations of CYS to culture media reduces the production of phenazine pigments in a range of type and clinical strains of *P. aeruginosa* without affecting growth. Fig. S3. Addition of sub-MIC concentrations of CYS to culture media reduces the release of pyomelanin pigment from these *B. cenocepacia* strains into the agar.

  PDF, 934K
