# Supplementary material for: Cysteamine, an Endogenous Aminothiol, and Cystamine, the Disulfide Product of Oxidation, Increase Pseudomonas aeruginosa Sensitivity to Reactive Oxygen and Nitrogen Species and Potentiate Therapeutic Antibiotics against Bacterial Infection
Source: Infect Immun. 2018 May 22;86(6):e00947-17. doi: 10.1128/IAI.00947-17 (PMC5964511; doi:10.1128/IAI.00947-17)
Supplement: Supplemental material [file IAI.00947-17_zii999092419s1.pdf]

Supplemental Data

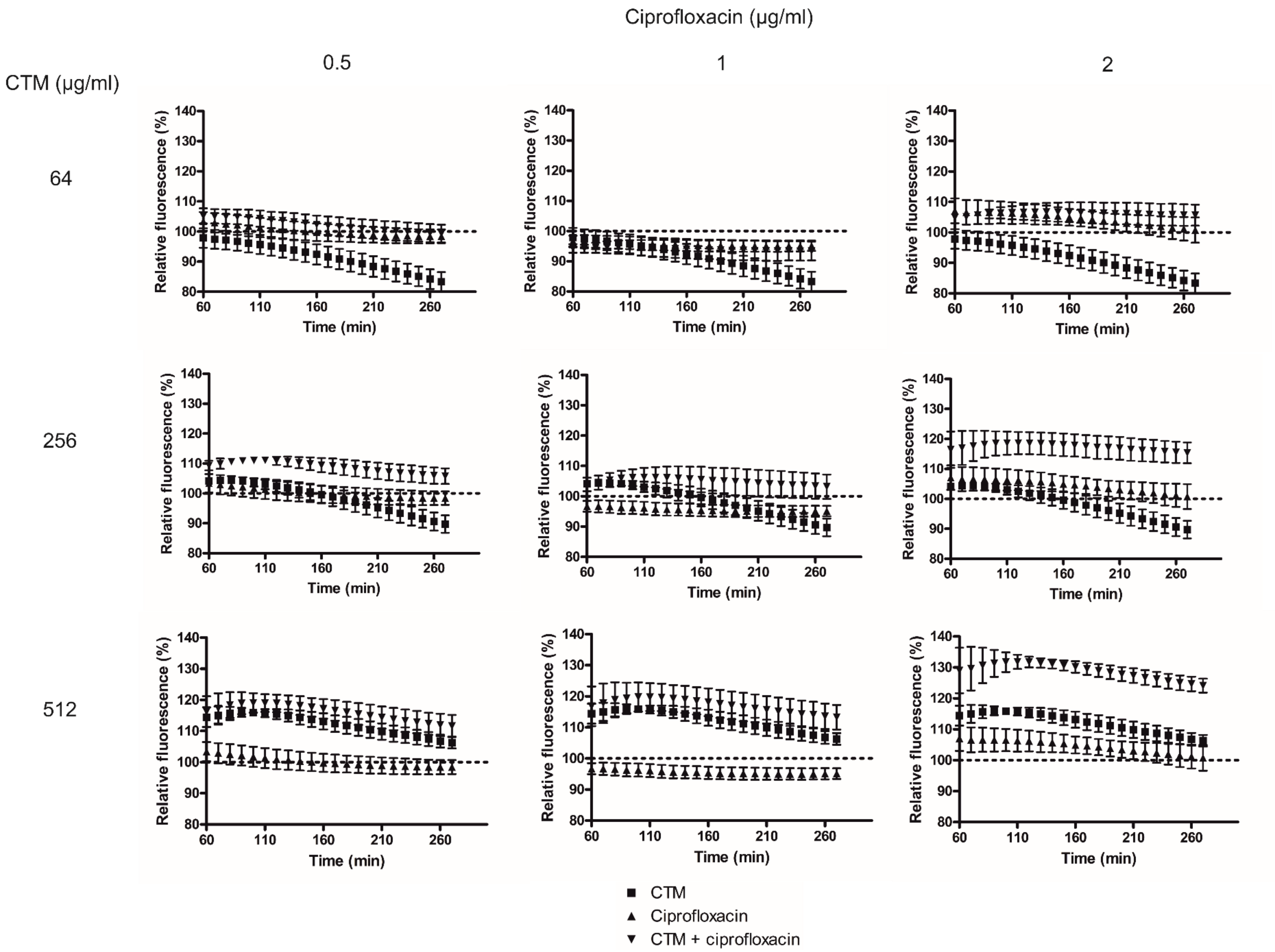
Ciprofloxacin (µg/ml)
0.5
1
2
CTM (µg/ml)
64
256
512
Relative fluorescence (%)
Time (min)
CTM
Ciprofloxacin
CTM + ciprofloxacin

Supplemental Figure 1. Relative H2DCFDA fluorescence of *Pseudomonas aeruginosa* PAO1 cultures treated with different combinations of ciprofloxacin and cystamine as shown over time compared to background fluorescence in media (dashed line) from which data at 2 h is analysed in Figure 2.

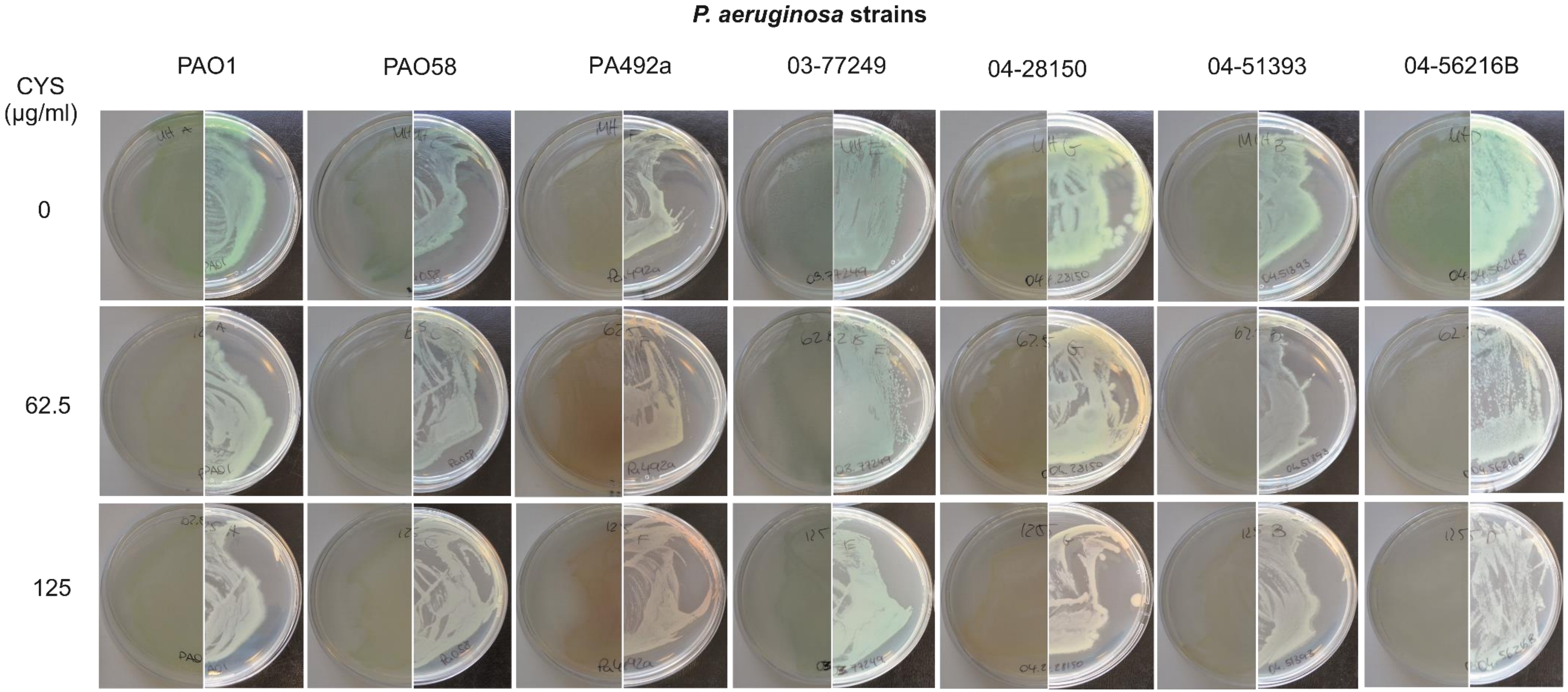

Supplemental Figure 2. Addition of sub-MIC concentrations of CYS to culture media reduces the production of phenazine pigments in a range of type and clinical strains of *P. aeruginosa* without affecting growth. Mueller-Hinton agar plates were supplemented with 0, 62.5 and 125 μg/ml of CYS as shown.

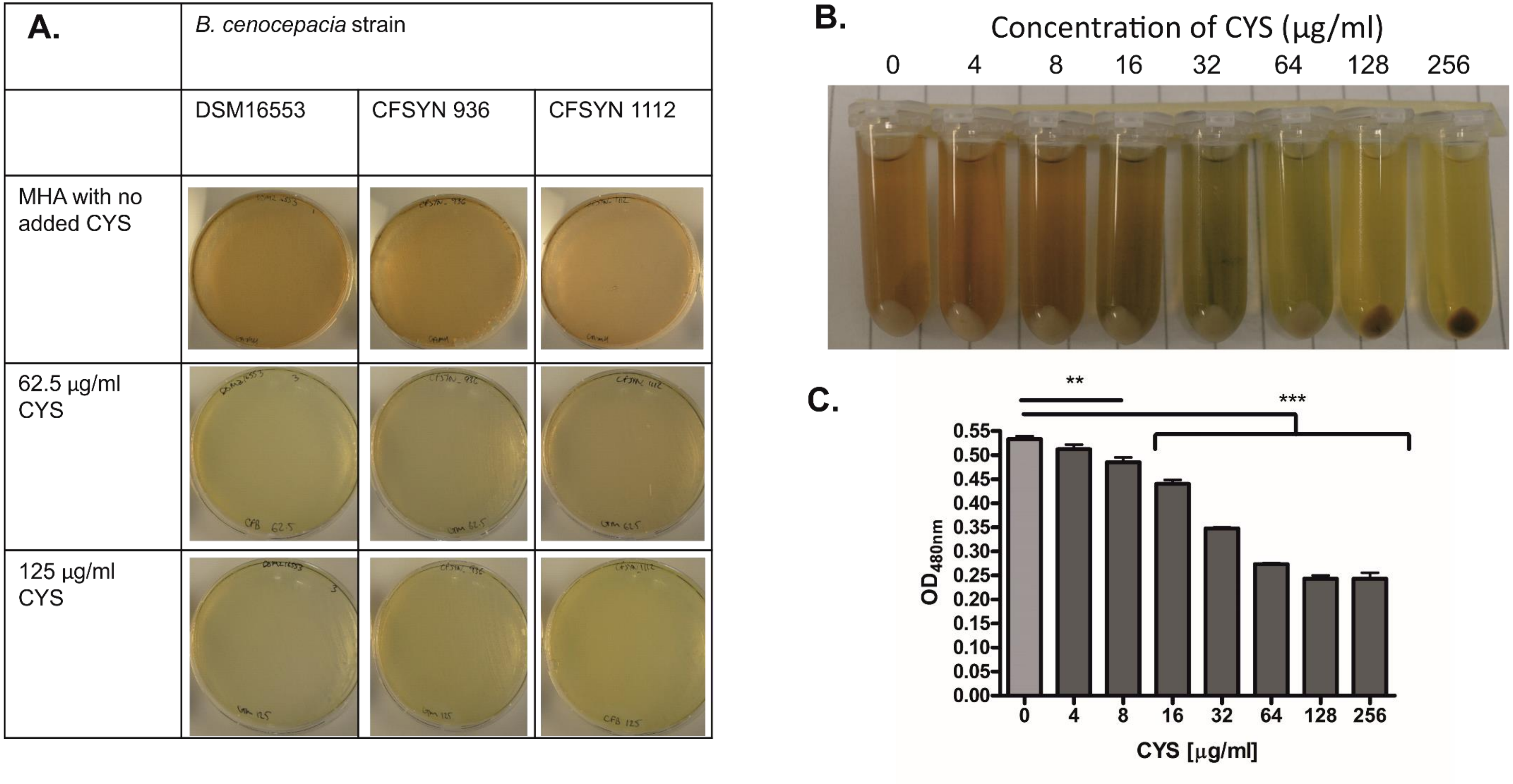

Supplemental Figure 3. Addition of sub-MIC concentrations of CYS to culture media reduces the release of pyomelanin pigment from these *B. cenocepacia* strains into the agar (**A**). DSM16553 was grown in Mueller-Hinton broth culture at 37°C for 20 h (**B**) and

exposed to a range of CYS concentrations as shown. CYS mediated an alteration in pigment production and release from the bacterial cell. Note the darkening of bacterial pellets following centrifugation at 5000 G for 5 min. Lysis in of the pellets in ethanol demonstrated an accumulation of intracellular pyomelanin at higher concentrations of CYS such as 128 µg/ml, but a significant reduction in pyomelanin detected by absorbance at 480 nm in the supernatant (C) was evident from concentrations of 8 µg/ml CYS and above (** = $p < 0.01$ *** = $p < 0.001$ One-way ANOVA, Tukey's post-test).
